# Nanostructured and Advanced Designs from Biomass and Mineral Residues: Multifunctional Biopolymer Hydrogels and Hybrid Films Reinforced with Exfoliated Mica Nanosheets

**DOI:** 10.1021/acsami.1c18911

**Published:** 2021-11-23

**Authors:** He Niu, Kaitao Zhang, Sami Myllymäki, Mostafa Y. Ismail, Paivo Kinnunen, Mirja Illikainen, Henrikki Liimatainen

**Affiliations:** † Fibre and Particle Engineering Research Unit, University of Oulu, P.O. Box 4300, FI-90570 Oulu, Finland; ‡ Microelectronics Research Unit, Faculty of Information and Electrical Engineering, University of Oulu, P. O. Box 4500, FI-90570 Oulu, Finland

**Keywords:** mechanochemistry, mica nanosheets, regenerated chitin, valorization, composites

## Abstract

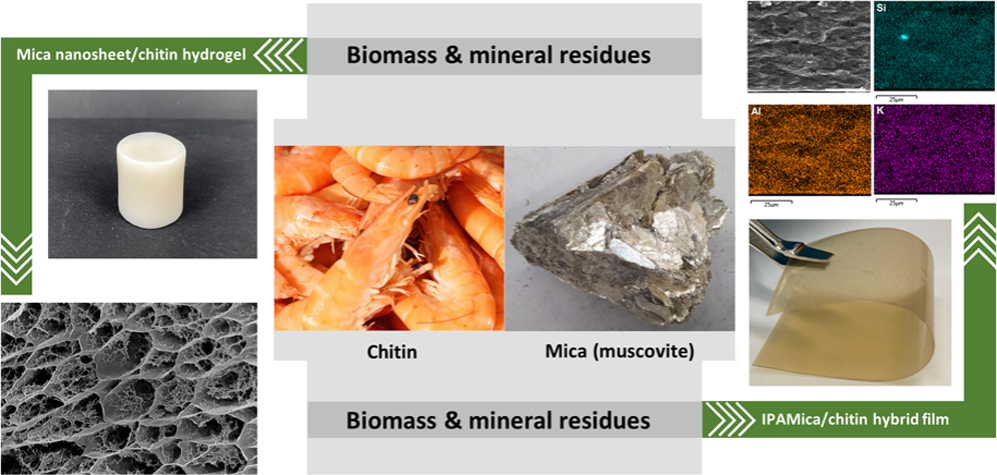

Transforming potential waste materials into high-value-added sustainable materials with advanced properties is one of the key targets of the emerging green circular economy. Natural mica (muscovite) is abundant in the mining industry, which is commonly regarded as a byproduct and gangue mineral flowing to waste rock and mine tailings. Similarly, chitin is the second-most abundant biomass resource on Earth after cellulose, extracted as a byproduct from the exoskeleton of crustaceans, fungal mycelia, and mushroom wastes. In this study, exfoliated mica nanosheets were individualized using a mechanochemical process and incorporated into regenerated chitin matrix through an alkali dissolution system (KOH/urea) to result in a multifunctional, hybrid hydrogel, and film design. The hydrogels displayed a hierarchical and open nanoporous structure comprising an enhanced, load-bearing double-cross-linked polymeric chitin network strengthened by mica nanosheets possessing high stiffness after high-temperature curing, while the hybrid films (HFs) exhibited favorable UV-shielding properties, optical transparency, and dielectric properties. These hybrid designs derived from industrial residues pave the way toward sustainable applications for many future purposes, such as wearable devices and tissue engineering/drug delivery.

## Introduction

Nanostructured composite and hybrid materials comprising nanoscale inorganic fillers and polymeric matrices have been investigated as wearable electronic devices or functional sensors.^1,2^ For example, montmorillonite, vermiculite, saponite, and kaolinite^3,4^ can be harnessed to fabricate layered silicate–polymer composites and to enhance polymer features without degrading its processability.^5^ Previously, composites based on layered silicates and petroleum-derived polymeric derivatives have been revealed, such as poly(vinyl alcohol) (PVA),^6^ poly(methyl methacrylate) (PMMA),^7^ epoxy resin,^8^ polystyrene (PS),^9^ polyimide (PI),^10^ and polypropylene (PP).^11^ However, the incorporation of nanoparticles with organic matrix, especially with natural and abundant polysaccharides, offers a route to design sustainable and high-value-added materials.^12^ So far, these advanced structures have mainly been synthesized from virgin, primary materials, or synthetic polymers, and realization of the full potential of industrial residues or byproducts in advanced hybrid materials has not been widely occurred.

Muscovite mica, the 2:1 layered silicate, is a well-known and abundant natural macroscopic and transparent inorganic crystalline mineral. Mica is a byproduct of the mining industry, and it can be recovered from mining waste rock and mine tailing concentrates through mineral processing.^13^ Therefore, it is a low-cost raw material and possesses many appealing properties, such as high light transparency, ultraviolet shielding, high dielectric constant, and high chemical and thermal stability.^14,15^ Natural mica typically exists as bulk material, and the form of thin sheets of mica is rare and can be obtained, e.g., using Scotch tape.^16^ Mica can also be converted to nanosheets using intercalation with polymers, microwave treatment, sonification, and combined physical and chemical methods,^17−20^ which expands the use of mica in several advanced applications. However, direct and straightforward mechanical grinding deteriorates the crystallinity of mica and alters its structural characteristics, and the exfoliation of mica still shows a significant challenge. In contrast, certain chemically assisted mechanochemical treatments display a feasible and efficient approach to individualize inorganic crystals.^21−24^ This methodology could also be used to liberate intact nanosheets from mica.

Chitin, a linear polymer of β-(1 → 4)-linked 2-acetamido-2-deoxy-β-d-glucose units, is the most abundant marine polysaccharide.^25^ It exists widely in the exoskeletons, i.e., shells and skeletal mantels of crustaceans, particularly shrimps, crabs, and lobsters, and large quantities of chitin-containing byproducts are created by the food-processing industry. For instance, the annual accumulation of waste marine shells is around six to eight million tons globally.^26^ Chitin denotes an interesting alternative to other biomaterials due to its physical and chemical stability, biodegradability, low toxicity, and biocompatibility with antimicrobial activity.^25,27^ Lately, advanced chitin-based materials were produced by the dissolution of chitin in alkaline KOH/urea solution.^28,29^ This method could be used to synthesize nanostructured artificial nacre-like structures with brick-and-mortar arrangements of organic and inorganic layers, yielding excellent strength and toughness.^30−35^ A one-step and robust synthesis route could provide an alternative manufacturing method for time-consuming layer-by-layer (LbL) alignment, etc.^36^ However, inorganic–organic systems based on mica nanosheets and regenerated chitin matrix, i.e., structures comprising solely of industrial side streams of mica and chitin, have not been widely addressed.

In this study, we propose a facile exfoliation method combining mechanochemical treatment and ultrasonication to obtain crystalline-delaminated mica nanosheets from natural mica, which is a byproduct of mining sites. The alkaline aqueous dispersion of mica nanosheets was used for the dissolution of biopolymeric chitin and further for the creation of nanostructured hybrid hydrogels and films of mica nanosheets and chitin matrix. Combining these industrial residues resulted in multifunctional and cross-linked hybrid designs with advanced mechanical, thermal, optical, and electric characteristics. Consequently, the developed approach fosters the beneficiation of inorganic and biomass industrial residues and wastes in high-value materials and exemplifies a potential waste-to-wealth route, according to the targets of the emerging green circular economy.

## Experimental Section

### Materials

Natural mica, i.e., muscovite, was obtained from Kaatiala, Finland (quartz as minor impurity). The mica rock was crushed and sieved to around 1 mm in TU-Clausthal, Germany. Raw chitin powder originating from shrimp shells was used without further treatment (Sigma-Aldrich; Germany). Potassium hydroxide (KOH), urea, epichlorohydrin (ECH), and lithium chloride (LiCl) were purchased from Sigma-Aldrich (Germany), and glycerol (99.5%) was obtained from VWR (Germany). All reagents were used as received. Deionized (DI) water was used throughout the experiments.

### Exfoliation of Mica to Nanosheets (IPAMica)

**Figure 1:**
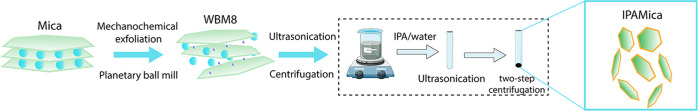
Schematic illustration of the mechanochemical exfoliation of mica using sequential planetary ball milling and ultrasonication in IPA/water to obtain mica nanosheets (IPAMica).

The exfoliation of mica was conducted using the mechanochemical method, followed by ultrasonication in 45 vol % isopropanol (IPA)/water solution^17^ to obtain mica nanosheets (IPAMica) (Figure 1). Initially, 5 g of as-received mica was planetary ball-milled at 175 rpm (Retsch PM200, Germany) in a 125 mL jar (8 milling balls with a diameter of 20 mm) in which 5 g of a solution of lithium chloride and methanol (40 wt %) was introduced as an exfoliation agent to obtain a wet ball-milled mica sample (WBM8, wet ball milling for 8 h). In contrast, DBM8 (dry ball mill for 8 h) was prepared as a reference without an exfoliation agent. WBM8 was filtered and washed with deionized water several times to remove any chemicals. Thereafter, the dry WBM8 was agate mortar-milled after being placed in a desiccator for 3 days. Then, 500 mg of the obtained powder was dispersed in 350 mL of 45% IPA/water solution, followed by ultrasonication for 20 min (400 W; 24 kHz). The IPAMica supernatant was obtained by centrifuging the previous suspension at 2000 rpm for 20 min to remove the unexfoliated WBM8. The supernatant was further centrifuged at 8700 rpm to obtain the IPAMica nanosheet, followed by repeated washing with deionized water by centrifuging to remove the isopropanol. Subsequently, the obtained IPAMica precipitate was freeze-dried for 3 days for future use.

### Preparation of Cross-Linked Hybrid IPAMica/Chitin Hydrogels

**Figure 2:**
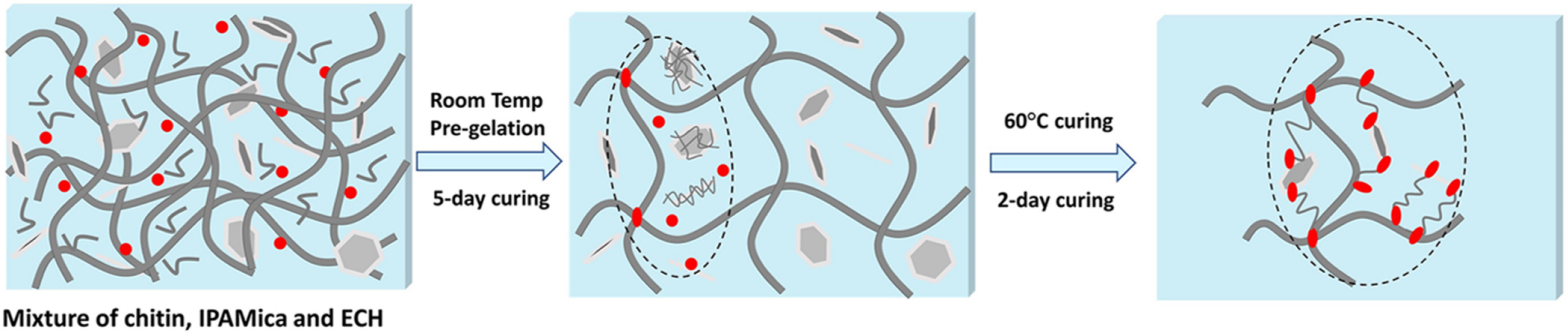
Schematic illustration showing the process for fabricating ECH cross-linked hybrid hydrogels of IPAMica and chitin (IPAMica/chitin/ECH). Red spheres illustrate the ECH cross-linker, light gray illustrates IPAMica plates, and dark gray strands illustrate the networked chitin structure.

First, 50 mg of WBM8 was dispersed in 35 mL of 45% IPA/DI-water solution to obtain the IPAMica, as mentioned above. The resulting IPAMica was redispersed in an aqueous 3.5 M KOH/0.6 M urea solution in a sonification bath. Then, chitin was dissolved in the alkaline solution and regenerated alongside IPAMica, as previously reported, with minor modifications.^29^ Briefly, the as-received chitin powder (3 wt % based on solution) was added to the solution with vigorous stirring under −30 °C for 15 min and further stirred at ambient temperature for 15 min. The process was repeated twice to obtain a homogeneous and viscous composite solution. Then, an ECH cross-linker was added to the mixture with a molar ratio of 1:2, 1:1, and 2:1 based on *N*-acetyl-d-glucosamine units of chitin. The mixture was transferred into a glass sample container and kept under ambient temperature for pregelation for 5 days prior to final curing in the oven at 60 °C for another 2 days (Figure 2). The obtained cross-linked hydrogels (referred to as IPAMica/chitin/ECH1:1, 1:2, and 2:1) were thoroughly washed with deionized water and further immersed in deionized water for 3 days to remove any chemical residues. The samples containing only chitin and IPAMica (without ECH cross-linker) and chitin with ECH (without IPAMica) were prepared as references.

### Preparation of Cross-Linked IPAMica/Chitin Hybrid Films (HFs)

**Figure 3:**
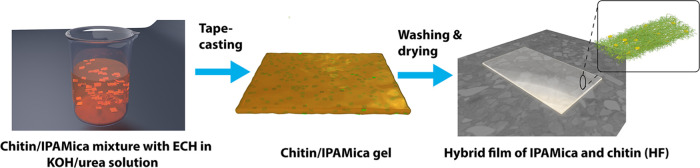
Schematic illustration showing the process for fabricating HFs of IPAMica and chitin via tape-casting.

The hybrid films of IPAMica and chitin were prepared using a similar process as hydrogels with minor modifications (Figure 3). First, the desired amounts of IPAMica (50, 100, 200, 400, and 800 mg) were dispersed into a 3.5 M KOH/0.6 M urea solution using sonification. Then, chitin powder (3 wt %; 1500 mg) was added to the solution with vigorous stirring at −30 °C for 15 min and was further stirred at ambient temperature for 15 min. The process was repeated twice to obtain a homogeneous and viscous solution. Then, ECH was added to the mixture with a molar ratio of 1:1 based on *N*-acetyl-d-glucosamine units of chitin. The solution was tape-casted on a polycarbonate plate after removing air bubbles and undissolved chitin by centrifugation at 8000 rpm for 20 min. The casted HF was kept at 4 °C for 40 h and then placed in 70% ethanol/water solution for regeneration for 2 h. Thereafter, the gelled mixture was stripped away from the plate and thoroughly washed with running water to remove residues. The obtained hybrid film was immersed in a 20 vol % glycerol/water solution for 30 min and cleaned before being fixed on the plastic plate by adhesive tape to dry at ambient temperature. Glycerol is used to enhance the ductility of HFs.^37^ The obtained HFs were named according to their IPAMica content (wt %) as 0.1, 0.2, 0.4, 0.8, and 1.6HF (based on 50 g of chitin solution). Also, a reference chitin film (CF) without any IPAMica was prepared (CF).

### Sample Characterizations

X-ray diffraction (XRD) analysis of the samples was conducted using a Rigaku SmartLab 4.5 kW (Japan) with the Co source (40 kV and 135 mA) Kα (Kα1 = 1.78892 Å; Kα2 = 1.79278 Å; Kα1/Kα2 = 0.5) at a scan rate of 3° min^–1^ and a step of 0.02°. Diffuse reflectance infrared Fourier transform spectroscopy (DRIFTS) spectra were obtained with a Bruker Vertex 80v spectrometer using 40 scans with a resolution of 1 cm^–1^. X-ray photoelectron spectroscopy (XPS) analysis was conducted using the Thermo Fisher Scientific ESCALAB 250Xi XPS System (U.K.). The binding energy (BE) adventitious carbon (C 1s = 284.8 eV) was used for the calibration. The samples were placed on an indium membrane with a passing energy of 20 eV and a spot size of 900 μm. Elements that induced silicon (Si), aluminum (Al), oxygen (O), potassium (K), and iron (Fe) were measured from the samples. The morphology of the samples was characterized by a field emission scanning electron microscopy (FESEM) (Zeiss ULTRA plus, Germany) with an acceleration voltage of 5 kV. Transmission electron microscope (TEM) images were obtained using TEM (JEOL JEM-2200FS, Japan) with an acceleration voltage of 200 kV. The ζ-potential and size distribution were measured using Zetasizer Pro (Malvern Panalytical, U.K.). Uniaxial compression tests were performed on the cross-linked hydrogels in their equilibrium state at ambient temperature, utilizing a Zwick Roell 10 kN machine (Germany). The cylindrical hydrogel with 20 mm in diameter and 10 mm in height was set on the lower plate and compressed by the upper plate at a strain rate of 1 mm min^–1^. Rheometry measurements of hydrogel were performed using a Q800 dynamic mechanical analyser (DMA) (TA instrument) and a 25 mm parallel plate geometry (Peltier plate steel) with an angular frequency of 1 rad s^–1^ and a loading gap of 2 mm using temperature ranges from 20 to 70 °C with an increasing step of 0.5 °C min^–1^. The transmittance (200–800 nm) of the HFs was measured using a UV–vis spectrophotometer (Shimadzu, Japan). Tensile tests were performed using a universal material testing machine (Instron 5544) equipped with a 100 N load cell. About three parallel specimens were cut into strips with 5 mm in width (thickness of 60 μm), and a 40 mm gauge length at a strain rate of 4 mm·min^–1^ was used. The dielectric permittivity and dielectric loss tangent of the film sample were measured using an Agilent E4991A impedance analyzer with a height of 60 μm at room temperature using short-open-load (LD-PE polymer 60 μm as a reference) calibration. During the measurements, ±10% height variation and permittivity variation were observed.

## Results and Discussion

### Exfoliation of Natural Mica Mineral

Lithium cation significantly affects dioctahedral mineral, such as muscovite, where previous research pointed out that lithium cation can both cation exchange with mica interlayer cations and occupy vacant sites in the octahedral layer^38−40^ and further affect the exfoliation of the minerals. Besides, some organic solvents can promote mechanochemical disintegration of layered minerals under wet conditions, dramatically increase the specific surface area, and enable retention of the crystallinity of the minerals.^21,41^ In this study, both dry and wet ball milling [in the presence of isopropanol (IPA)/water solution] were investigated as the first step of consequent milling-ultrasonication exfoliation of mica to IPAMica nanosheets. The dry and wet ball-milled powders (DBM8 and WBM8, respectively) exhibited rather different colors after milling treatment, DBM8 being gray while WBM8 turned yellowish (Supporting Information Figure S1a,b). This phenomenon has also been observed in other solvent-assisted mechanochemistry.^42^ Furthermore, DBM8 and WBM8 displayed significantly different morphologies. DBM8 comprised more irregular and spherical particles (the smallest entities having dimensions of hundreds of nanometers) having a fractal-like structure, while WBM8 contained nano- and microscale-layered flakes that were partly stacked together, as visualized using FESEM (Supporting Information Figure S1c,d). Due to the structural distortion and plastic deformation of dry powder during dry ball milling (DBM8), the particles tended to agglomerate and amorphized, leading to the collapse of the crystalline structure. WBM8 was delaminated to nano- and microsheets retaining its crystallinity, by contrast, resulting from Li^+^ exfoliation mechanism and methanol, which covered laminar particles as a lubricant. Large aggregates of smaller particulates were observed in both specimens; however, only WBM8 could generate layered nanosheets (IPAMica) after further ultrasonication and centrifugation.

**Figure 4:**
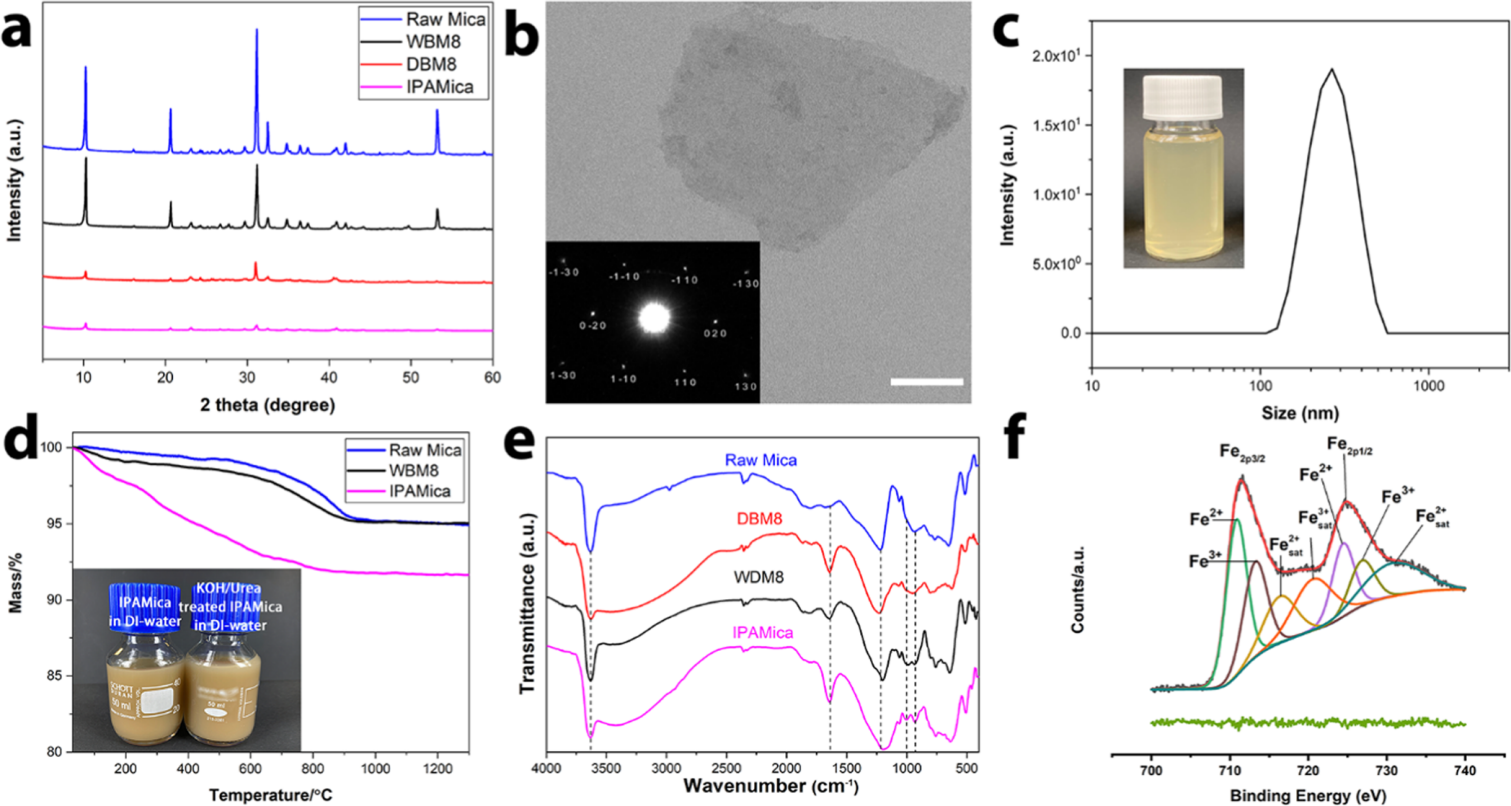
Characterization of mica samples. XRD patterns of raw mica, dry ball-milled mica, wet ball-milled mica, and IPAMica nanosheet (a). Single-layer mica nanosheet at low resolution with corresponding selected area electron diffraction (SAED) patterns indicating (001) direction (scale bar, 100 nm) (b). DLS analysis of the size of IPAMica nanosheets in 45% IPA/water solution (c). Thermogravimetric analysis (TGA) curves of raw mica, wet ball-milled mica, and IPAMica nanosheet; the digital photo displays DI-water dispersion of IPAMica with 2 mg·mL^–1^ (left) and redispersion after KOH/urea low-temperature treatment (right) (d). DRIFT spectra of raw mica, dry ball-milled mica, wet ball-milled mica, and IPAMica nanosheet (e). Deconvolution of XPS spectrum for wet ball-milled mica (f).

XRD patterns of ground samples show that WBM8 retained a crystalline structure similar to that of raw mica, while DBM8 became partially amorphous (Figure 4a), which parallels the observation in SEM analysis. Particularly, the diffraction peak (001) at around 2 theta of 10° decreased for the IPAMica nanosheet, which indicated that delamination of the layered structure occurred along this lattice plane.^43^ The exfoliation along the cleavage plane (001) was confirmed by the selected area diffraction pattern (SAD) of the monolayer nanosheet.^44^Figure 4b displays the morphology of monolayered IPAMica nanosheets, while the bulk IPAMica is illustrated in Supporting Information Figure S2a. The observed diameter of IPAMica nanosheets ranged from 200 to 400 nm, which paralleled the particle size obtained using dynamic laser scattering (DLS) (Figure 4c). Furthermore, high-resolution TEM (HRTEM) images of a single-layer mica nanosheet indicate the spacing of lattice planes to be around 0.45 nm (Supporting Information Figure S2b). This space is associated with lattice planes (11) and (02).^18,45^

IPAMica can homogeneously be dispersed in DI-water, and no significant change was observed on redispersed IPAMica in DI-water after treatment with KOH/urea solution at low temperature (Supporting Information Figure S2c). The stability of IPAMica in DI-water was evidenced by a constant ζ-potential value (−17.82 mV). Furthermore, IPAMica showed quite different thermal behavior than raw mica and WBM8 (Figure 4d). IPAMica displayed a continuous and almost linear mass loss until 800 °C. In contrast, only conspicuous dehydroxylation occurred at around 800–900 °C with raw mica and WBM8. This difference may occur because of the large specific surface area of mica nanosheets, which enhances the absorption of surrounding water molecules.

DRIFT spectra display the alteration of silicon–oxygen stretching vibration modes around the 1100–1000 cm^–1^ region (Figure 4e), which is related to the particle size, as the thickness of the crystals is thinner than 100 nm.^46^ The band observation of the conspicuous OH bending frequency at around 934 cm^–1^ may display a more firmly bonded hydroxyl due to three octahedral ions (2 Al, 1 Li) nearby in the mica nanosheet compared with the original two octahedral sites (2 Al, 1 void). Besides, a decrease in OH bending frequency can be seen from 938 to 936 cm^–1^ and to 934 cm^–1^ with raw mica, WBM8, and IPAMica, respectively, indicating that the lithium ions enter the empty sites in the dioctahedral layer.^38^ Consequently, this migration alters the overall layer charge due to excessive positive cations, thereby expelling the K^+^ in the interlayer. The H–O–H bending band around 1640 cm^–1^ is assigned to the adsorbed water (δ(H_2_O) vibration). A weak peak was observed in raw mica; however, a very strong band is seen in IPAMica,^47^ which is in accordance with the thermal data (Figure 4d).

The XPS Fe_2p_ spectrum of WBM8 (Figure 4f) shows two orbital splits as 2p_1/2_ and 2p_3/2_ states, in which the deconvolution of the Fe_2p_3/2__ results in two main components: Fe(II) at BE = 710.8 eV with a correlated satellite peak at BE = 716.3 eV and Fe(II) at BE = 713.2 eV with a correlated satellite peak at BE = 720.6 eV, which is similar to the literature.^48^ The binding energy of the Fe(II) 2p_3/2_ peak increases from raw mica to WBM8, which can result from a loss of a ligand (oxygen in mica) for Fe(II).^49^ Previous research has shown that the structural environment and crystal field effect significantly affect muscovite’s color.^50^ Therefore, the yellowish color of WBM8 could result from the increase in the binding energy of Fe(III) and Fe(II) (Supporting Information Table S1). The reduction of Fe(III) to Fe(II) in octahedral sites can alter the mica crystal structure, thereby increasing the swelling of the clay and facilitating the exfoliation of WBM8.^51^ Fe(III) content increases from raw mica to DBM8, showing the iron oxidation in DBM8 (Supporting Information Figures S3 and S4). XPS spectra for Si_2p_ show a relatively stable bond environment (Supporting Information Figure S5 and Table S2). The binding energy for raw mica (BE = 102.6 eV) and for both ground samples (BE = 102.5 eV) coincided with the tetrahedral coordination close to the reported literature of muscovite.^52,53^ Once the Al_2p_ binding energy is combined, it reveals that the inherent structure is retained after wet ball milling. In contrast, partial amorphization occurred for DBM8, causing decreased crystallinity. DBM8 obtained the highest binding energy of K, while the lowest binding energy was observed for WBM8. This phenomenon is likely due to the overall contribution from iron states in ground mica in which DBM8 possesses a higher content of Fe(III) components. Apart from the effect of the iron state, Li^+^ cations either occupy the voids in octahedral layers or cation exchange with K^+^, further influencing the binding energy of potassium.

### Cross-Linked IPAMica/Chitin Hybrid Hydrogels

**Figure 5:**
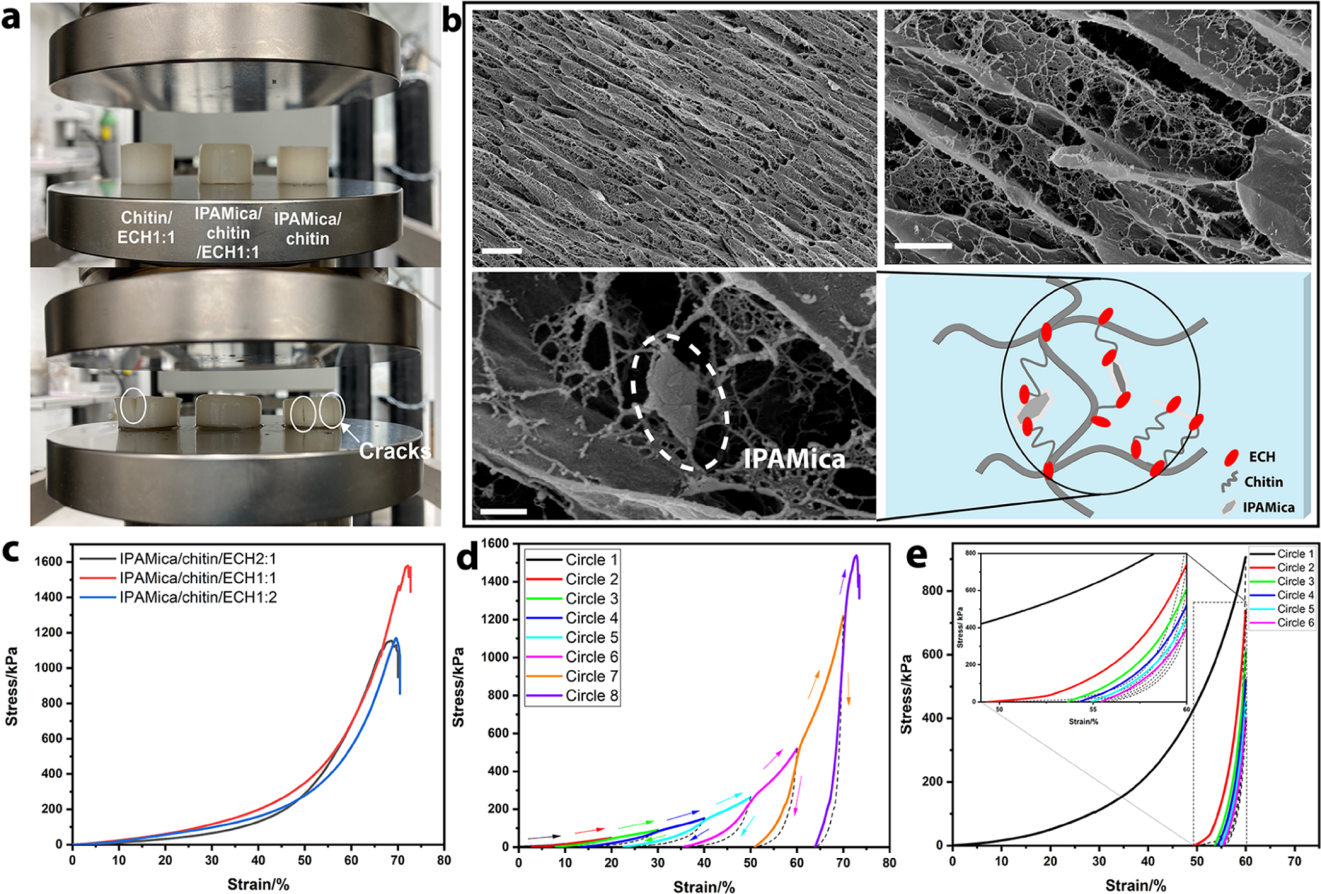
Characterization of IPAMica/chitin hydrogels. Digital image of the chitin/ECH, IPAMica/chitin/ECH, and IPAMica/chitin hydrogels after 40% strain deformation (a). SEM images of cross-sectional structures of IPAMica/chitin/ECH1:1 (scale bar from left to right, 10 μm, 2 μm, and 300 nm) (b). Compressive stress–strain curves of IPAMica/chitin/ECH hybrid hydrogels with different molar ratios of ECH (c). Compressive stress–strain curves of IPAMica/chitin/ECH1:1 during loading–unloading cycles until failure (d). Fatigue behavior of IPAMica/chitin/ECH1:1 hybrid hydrogel at 60% strain (e).

The alkaline medium of KOH/urea was used to disperse IPAMica nanosheets and further dissolve chitin in the presence of an ECH cross-linker to obtain nanostructured hybrid hydrogels after gelatinization (Figure 2). It has been reported that nanoparticles can be used as physical cross-linkers for the formation of hydrogels based on polysaccharides, such as cellulose.^54,55^ This cross-linking is mediated via polymer–particle interactions, ionic bonds, and hydrogen bonds. Here, the negatively charged mica nanosheets can be linked with cationic chitin via electrostatic interactions. Moreover, the mica nanosheets possess hydroxyl groups on the edges of octahedral layers, which can further react with an ECH cross-linker to create ether bridges with chitin chains.^56^ Therefore, IPAMica acted as both a physical cross-linker in the chitin polymeric network and facilitated chemical cross-linking in the hydrogels. Double-cross-linked hydrogels (IPAMica/chitin/ECH) presented superior compressive behavior compared to the references that were either physically (IPAMica/chitin) or chemically cross-linked (chitin/ECH) (Figure 5a). However, a relatively long gelation reaction was conducted using both pregelation and heat curing for 7 days in total (Supporting Information Figure S6a), indicating a slower cross-linking rate of hybrids compared with previous studies.^56−58^ The suitable gelation (curing) temperature was confirmed by the curve of temperature dependence of storage modulus (*G*′) and loss modulus (*G*″), where a cross point of *G*′ and *G*″ was noticed at around 60 °C (Supporting Information Figure S6b).

The hierarchical and interconnected structure of porous hydrogels was revealed using FESEM (Figure 5b) to illustrate the design that comprised larger strands of chitin linked by IPAMica nanosheets and smaller chitin fibrils. The structure contained both nano- and microsized irregular and connected open pores (Supporting Information Figure S7a). The IPAMica nanosheets were bound in the network mainly from the edges of the sheets, where the hydroxyl group is located. Besides, there existed polymeric nanospheres, which were smaller than previously reported for regenerated cellulose (about 200 nm) (Supporting Information Figure S7b).^59^ Compared to the hybrid hydrogels of IPAMica/chitin/ECH, both the reference hydrogels (IPAMica/chitin and chitin/ECH representing the physically or chemically cross-linked hydrogels) presented a more uniform assembly with less microstructural hierarchy and contained mainly micropores of a smaller size. In addition, the physically cross-linked hydrogel expressed a less uniform ordering and a thicker wall of regenerated chitin compared to the chemical cross-linked one (Supporting Information Figure S8).

Both physically and chemically cross-linked reference hydrogels exhibited easier rupture when loaded to 40% strain deformation, and clear cracks appeared on the side surface of the hydrogels, while the hybrid hydrogel remained intact (IPAMica/chitin/ECH1:1). The stress at fracture of hybrid hydrogels reached a maximum value of 1.57 MPa with a fracture strain of 72% at an ECH-to-*N*-acetyl-d-glucosamine unit molar ratio of 1:1 (IPAMica/chitin/ECH1:1). The corresponding values of the ECH molar ratio of 2:1 and 1:2 were 1.15 MPa at 68% and 1.17 MPa at 69%, respectively (Figure 5c). Figure 5d,e shows the high stiffness, where energy was absorbed by the double-cross-linked structure, which provided high load-bearing performance for the hydrogel.^58^ There was apparent plastic deformation and strength degradation during the loading–unloading cyclic test with a set of 60% strain. This irreversible deformation can be attributed to the physically cross-linked IPAMica nanosheets and chitin nanospheres within the micropores. However, these structures could transfer the mechanical stress across the connected chitin network and dissipate the energy, preventing full fracture of the hydrogel during the cyclic compressive test. Therefore, mica nanosheets show their potential as physical cross-linkers that affect the characteristics of the final hydrogel. The hydrogel properties can further be adjusted by controlling the gelation process, thereby fabricating hybrid hydrogels with the desired mechanical properties. For example, the hybrid hydrogel showed network stiffening when stored at room temperature for 2 months (Supporting Information Figure S9). The properties of clay-chitin/chitosan systems are summarized in Supporting Information Table S3, highlighting the good performance of IPAMica/chitin/ECH hydrogels.

### Cross-Linked IPAMica/Chitin HFs

**Figure 6:**
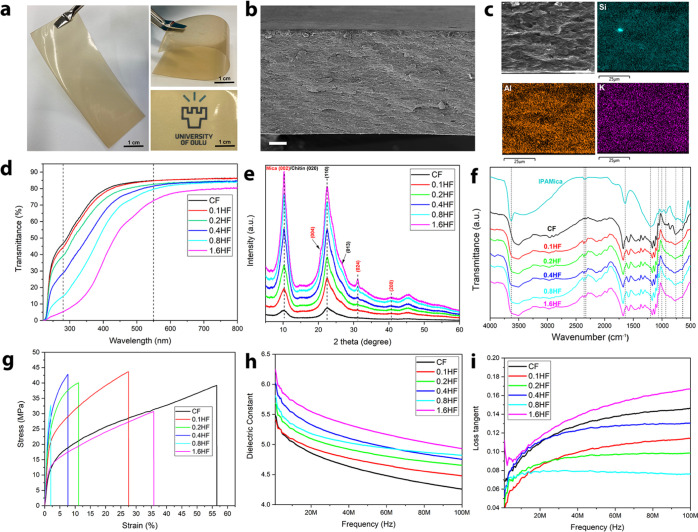
Characterization of HFs. The digital photo displays the flexible and transparent appearance of the IPAMica/chitin HF (1.6HF) (a). SEM image of the cross section of 1.6HF (scale bar, 10 μm) (b). Homogeneous distribution of IPAMica nanosheet in chitin matrix indicated by elemental mapping using EDX (c). UV–visible light transmittance (∼60 μm thickness) (d). XRD patterns (e) and DRIFT spectra (f). Representative stress–strain curves (g), and frequency dependency of the dielectric constant (h) and loss tangent of chitin film (CF) and IPAMica/Chitin HFs (i).

Self-standing HF with different IPAMica contents from 0.1 to 1.6 wt % was prepared by dissolving chitin in alkaline dispersion of IPAMica nanosheets, followed by tape-casting, washing, and drying (Figure 3). This robust and straightforward approach can easily be adapted at larger-scale film fabrication, and the technique is more efficient than, for example, a typical layer-by-layer process in which hundreds of layers are typically required to reach a thickness above 1.5 μm.^32^ The HFs possessed a transparent and yellowish appearance depending on the content of the IPAMica, and 1.6HF was highly flexible when bent (Figure 6a and Supporting Information Figure S10). FESEM with energy-dispersive X-ray analysis (EDX) displays the morphology of 1.6HF (Figure 6b,c). The mica nanosheets are homogeneously aligned among the regenerated chitin matrix, analogously to the monolithic mortar-like structure. The cross-sectional SEM images of all HFs with variable IPAMica content are given in Supporting Information Figure S11, showing a homogeneous and partly layered ordering of IPAMica in the chitin matrix without obvious cracks or voids. A larger magnification of the cross section of the 1.6HF clearly shows the planar orientation of the nanosheets and displays that the sheets were incorporated in the matrix without breakage, as described by the “pull-out” model (Supporting Information Figure S12).^33^ UV–visible spectra reflect the transparency of the HFs in the visible range (approximately 500–550 nm) and UV-blocking capability at low wavelengths (<280 nm) (Figure 6e). A noticeable decrease of transmittance at the UV range is achieved with 1.6HF (UV transmittance of approximately 5%) compared with that of the original chitin film (47%), while only a slight reduction in transmittance can be seen in the visible region (from 84 to 73% for CF and 1.6HF, respectively).

The XRD and DRIFT spectra reflected the coexistence of IPAMica and the regenerated chitin matrix (Figure 6e,f). A higher crystallinity was observed with an increase in IPAMica content. Nevertheless, the polymer matrix still attains the pronounced domination of the background spectra. The main XRD peaks of mica overlap with those of regenerated chitin, in which (002) and (004) reflections of mica overlay with (020) and (110) reflections of chitin. The shoulder peaks and humps, such as (004), (024), and (200) were observed with the incremental IPAMica content. A similar trend can be seen from DRIFT spectra, where the band of hydroxyl stretching shifts to a higher wavenumber as the incremental content of the nanosheets. The band at 2362 cm^–1^ becomes noticeable in 1.6HF compared with CF, which is also observed in the original IPAMica. Due to the embedment of IPAMica, the band ranging from 900 to 1100 cm^–1^ becomes more complex. For instance, the bands at 1063 and 997 cm^–1^ are assigned to *v*_as_(Si–O) and *v*(Si–O–Si) in mica, which overlaps with the amide III and C–O–C groups of chitin.^47,60^ In addition, the band of the N–H group around 650–750 cm^–1^ is strongly influenced by δ(Si–O–T) and *v*(Al–O–Al) of mica.^61,62^

Figure 6g shows the alteration of mechanical characteristics of HFs as a function of IPAMica content. The IPAMica nanosheets reinforced the HFs by enhancing the stiffness (from 0.851 to 3.537 GPa) and tensile strength (from 39.21 to 43.69 MPa) of the films, while the strain of the films decreases. A larger proportion (>0.4 wt %) of mica is detrimental for the film strength, as the nanosheets may provide weak spots for crack propagation, presumably due to the limited adhesion between the IPAMica and matrix. Moreover, microscopic air bubbles could be firmly attached to nanosheet surfaces, causing a large volume of invisible pores even after centrifugation pretreatment. This is a typical behavior of biopolymeric films reinforced with nanoparticles, and strength decrease with increasing fraction of nanoparticles has also been reported in other systems, such as alumina/chitosan.^31,34,35^ When the loading of IPAMica increases to 1.6HF, the strain at break becomes 37.93%, which is almost 38 times larger than that of 0.8HF (0.99%). Since the amount of IPAMica in 1.6HF is doubled that of 0.8HF, the hydrogen bonds between IPAMica and chitin fibrils participate in the load-carrying phase and dissipate stress during deformation, showing an enhanced toughness. Supporting Information Table S4 summarizes the overall mechanical properties of HFs.

Mica is a natural electrical insulation material due to its intrinsic high dielectric property, and it can be used in the fabrication of hybrid materials for flexible and transparent electronic devices, such as soft robotics, light-emitting diodes, and wearable electronics.^63,64^ The dielectric properties of HF regarding real permittivity and dielectric loss tangent are presented in the frequency range from 1 to 100 MHz in Figure 6h,i, respectively. The dielectric permittivity gradually increased as a function of IPAMica content (approximately from 4.6 to 5.3 at the middle of the measured frequency range), while the dielectric loss tangent was noted to be significantly lower above 30 MHz (except with 1.6HF) when the nanosheets were embedded in the films. 1.6HF exhibits equivalent permittivity as mica/nanofiber cellulose film at a low frequency of 1 MHz.^65^ Consequently, the permittivity frequency relationship decreases and the loss tangent increases at higher frequencies, as expected. Overall, 0.8HF exhibits a favorable dielectric performance regarding dielectric permittivity and loss at a relatively high frequency at 100 MHz.

## Conclusions

In summary, crystalline and delaminated mica nanosheets with a high aspect ratio were successfully obtained using the mechanochemical exfoliation of natural mica, which is a byproduct from mining sites. Nanostructured and multifunctional hydrogels and self-standing films of regenerated chitin, an abundant biopolymeric residue, reinforced with exfoliated mica nanosheets were further fabricated from the alkaline dispersion of nanosheets through a robust and straightforward procedure. The hybrid hydrogels displayed a hierarchical and open nanoporous structure consisting of an enhanced load-bearing double-cross-linked polymeric chitin network strengthened by mica nanosheets possessing high stiffness after high-temperature curing. Due to the intrinsic properties of nanosized mica, the HFs exhibited enhanced electric permittivity and favorable optical properties with ultraviolet blocking performance while retaining high transparency at visible wavelengths. A small proportion of nanosheets enhanced the stiffness and tensile strength of the films, while a slight deterioration of mechanical properties was seen at high dosages, potentially resulting from the poor matrix–aggregate interaction. These biocompatible and environmentally friendly hybrid designs derived from inorganic and biomass industrial residues could be applied in many potentially advanced applications such as wearable devices and tissue engineering/drug delivery purposes.

## Supporting Information Available

Supporting data including digital photo and SEM images of DBM8 and WBM8; high-resolution TEM images and ζ-potential of mica nanosheets; XPS analysis of raw mica, DBM8, and WBM8; digital photo and temperature-dependent rheological behavior of pregelated hydrogel; SEM images of IPAMica/chitin hydrogel; cross-sectional SEM images of physically cross-linked/chemically cross-linked/double-cross-linked hydrogels; digital photo of chemically cross-linked/double-cross-linked hydrogels for 2 months under room temperature; comparison table of different clay-chitin/chitosan-based hydrogels in the literature; digital photo of chitin film and hybrid films; cross-sectional SEM images of hybrid films; and a summary table of mechanical properties of hybrid films (PDF)
