# Supplementary material for: Nanostructured and Advanced Designs from Biomass and Mineral Residues: Multifunctional Biopolymer Hydrogels and Hybrid Films Reinforced with Exfoliated Mica Nanosheets
Source: ACS Appl Mater Interfaces. 2021 Nov 23;13(48):57841–50. doi: 10.1021/acsami.1c18911 (PMC8662632; doi:10.1021/acsami.1c18911)
Supplement: Supplementary file 1 — am1c18911_si_001.pdf [file am1c18911_si_001.pdf]

Supporting Information

Nanostructured and Advanced Designs from Biomass and Mineral Residues: Multifunctional Biopolymer Hydrogels and Hybrid Films Reinforced with Exfoliated Mica Nanosheets

He Niu[†]*, Kaitao Zhang*[†]*, Sami Myllymäki*[‡]*, Mostafa Y. Ismail*[†]*, Paivo Kinnunen*[†]*, Mirja Illikainen*[†]*, Henrikki Liimatainen*[†]*

[†] Fibre and Particle Engineering Research Unit, University of Oulu, P.O. Box 4300, FI-90570 Oulu, Finland

[‡] Microelectronics Research Unit, Faculty of Information and Electrical Engineering, University of Oulu, P. O. Box 4500, FI-90570 Oulu, Finland

* Corresponding author: Henrikki Liimatainen

Email: Henrikki.Liimatainen@oulu.fi Tel.: +358 8553 2416; Fax: +358 8553 2405.

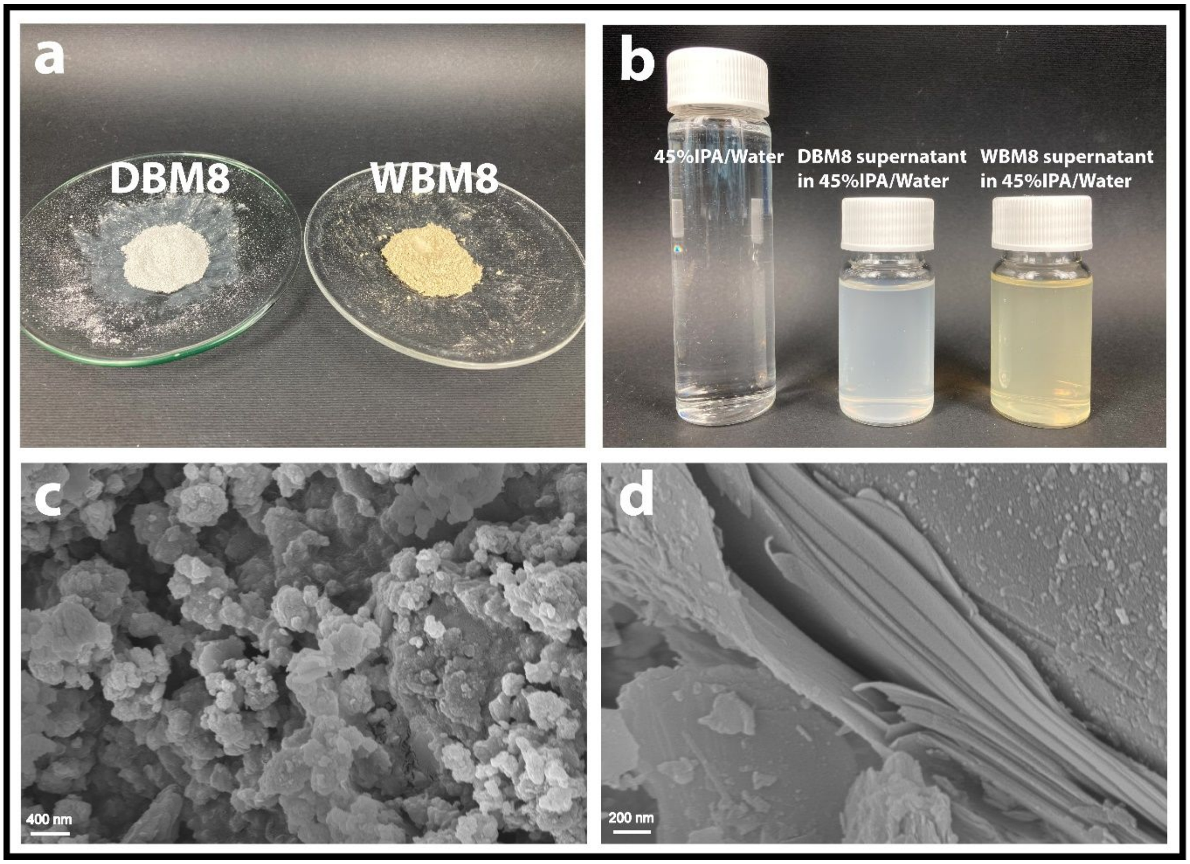

Figure S1. Digital photo of DBM8 (grey powder) and WBM8 (yellow powder) (**a**), IPAMica supernatant after ultrasonication and centrifugation for DBM8 and WBM8 (**b**), and SEM images of DBM8 (**c**) and WBM8 (**d**), respectively

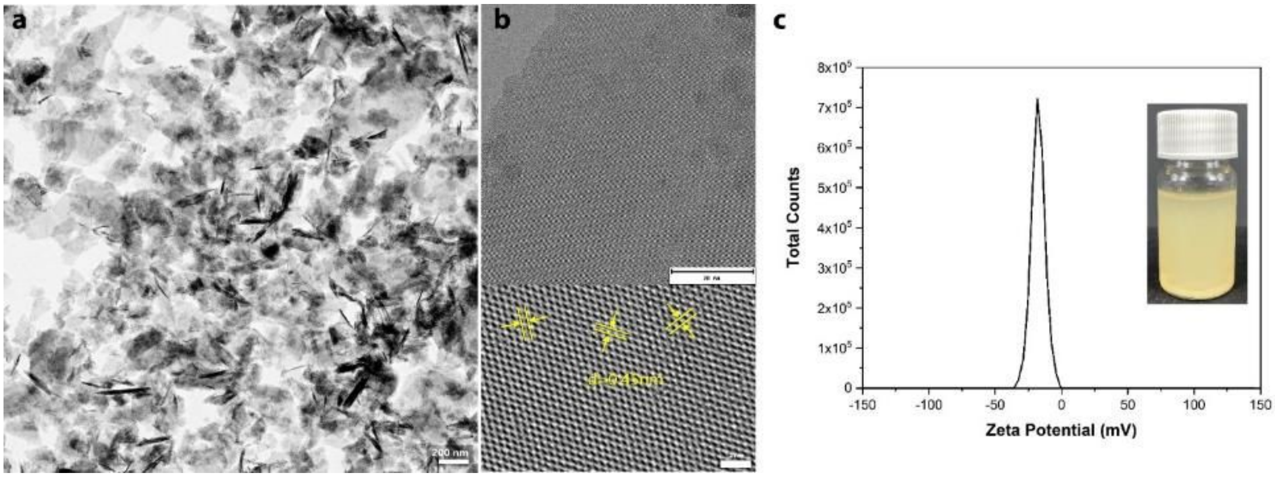

Figure S2. TEM images of bulk IPAMica morphology (**a**), high-resolution TEM images of a single-layer mica sheet (**b**), zeta potential of IPAMica in DI-water (**c**)

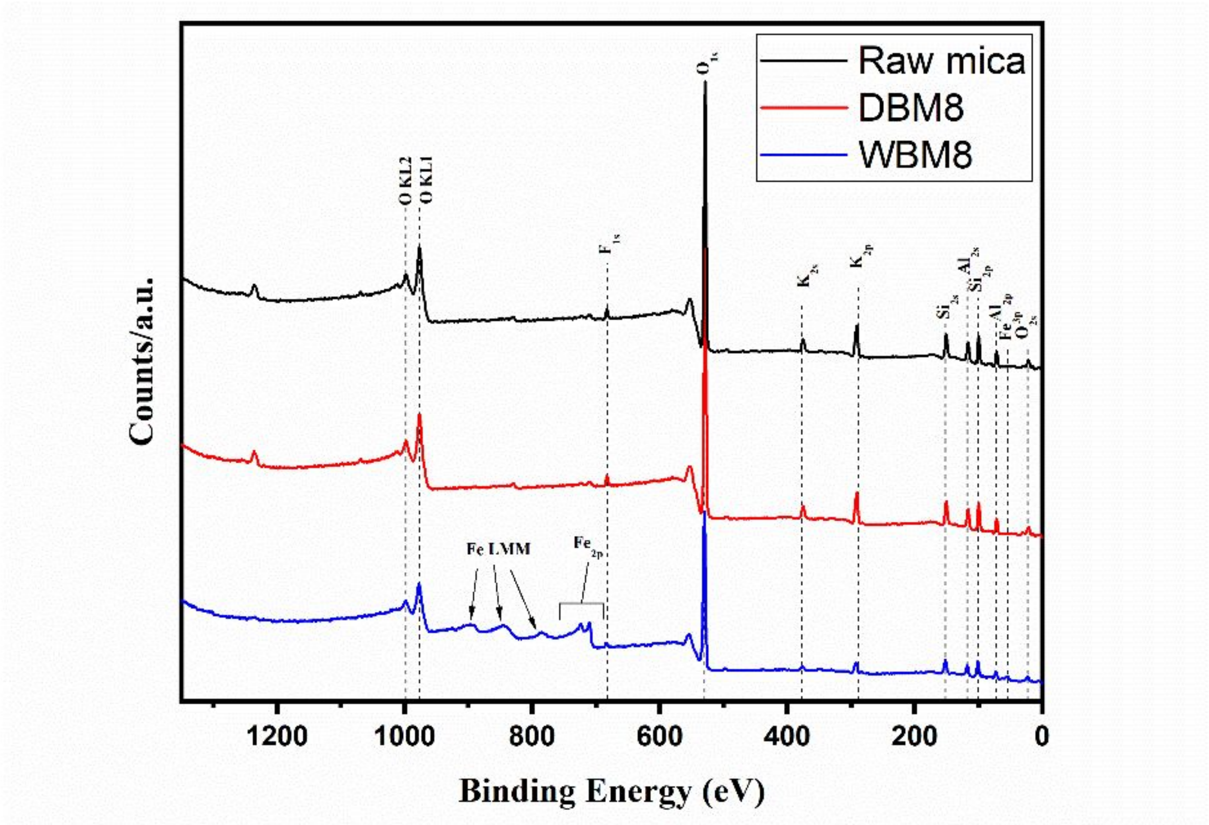

Figure S3. XPS spectra of raw mica, DBM8, and WBM8

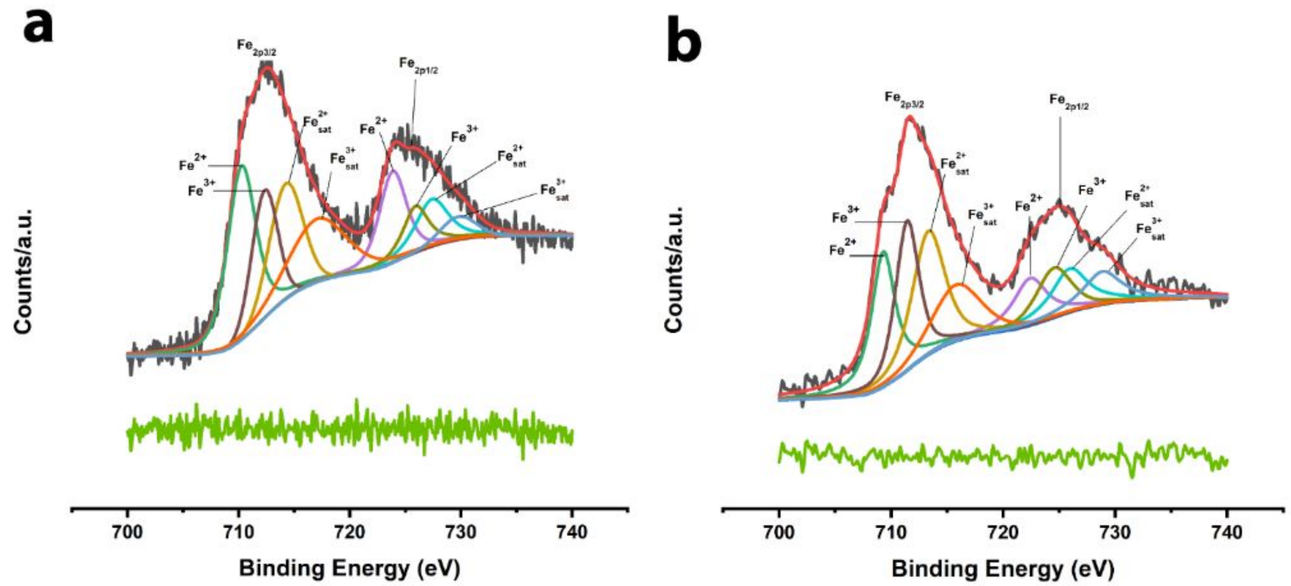

Figure S4. XPS Fe 2p spectra for raw mica (**a**), and DBM8 (**b**)

Table S1 Detailed Fe_{2p}-binding energy (in eV) for raw mica, DBM8, and WBM8

Sample name	Peak position		
	Assignment	Fe $2p_{3/2}$	Fe $2p_{1/2}$
Raw mica	BE Fe^{2+}	710.3 eV	723.8 eV
	BE Fe^{2+}_{sat}	714.2 eV	727.7 eV
	BE Fe^{3+}	712.3 eV	725.9 eV
	BE Fe^{3+}_{sat}	717.1 eV	729.8 eV
DBM8	BE Fe^{2+}	709.2 eV	722.4 eV
	BE Fe^{2+}_{sat}	713.3 eV	725.9 eV
	BE Fe^{3+}	711.4 eV	724.5 eV
	BE Fe^{3+}_{sat}	715.8 eV	728.9 eV
WBM8	BE Fe^{2+}	710.8 eV	724.4 eV
	BE Fe^{2+}_{sat}	716.3 eV	730.7 eV
	BE Fe^{3+}	713.2 eV	726.8 eV
	BE Fe^{3+}_{sat}	720.6 eV	-

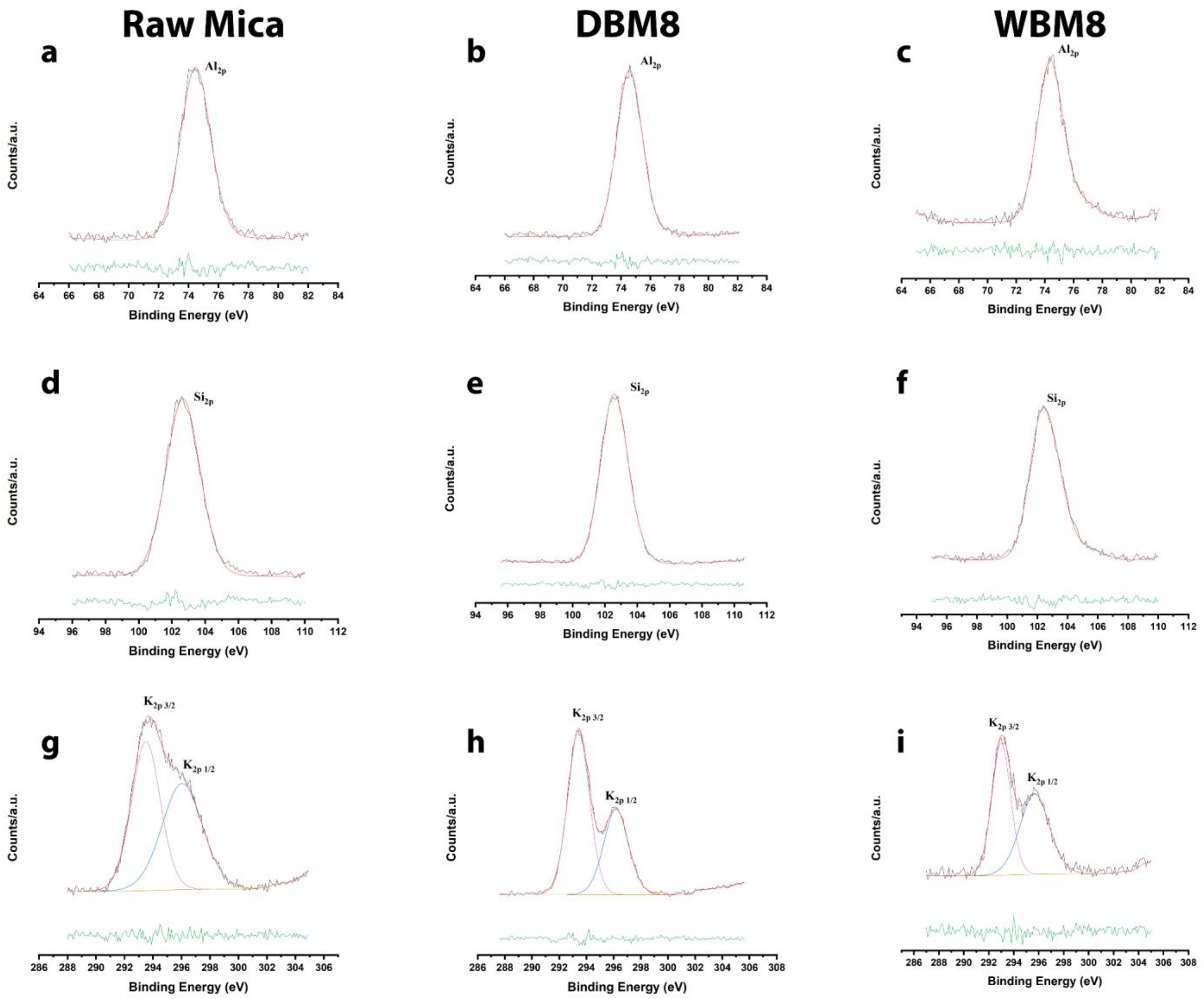

Figure S5. Al_{2p}, Si_{2p} and K_{2p} XPS spectra of raw mica, DBM8, and WBM8

Table S2 Detailed Si_{2p}, Al_{2p} and $K_{2p3/2}$-binding energy (in eV) for raw mica, DBM8, and WBM8

Sample name	Peak position		
	BE Si_{2p}	BE Al_{2p}	BE $K_{2p3/2}$
Raw Mica	102.6 eV	74.3 eV	293.4 eV
DBM8	102.5 eV	74.6 eV	294.1 eV
WBM8	102.5 eV	74.0 eV	293.0 eV

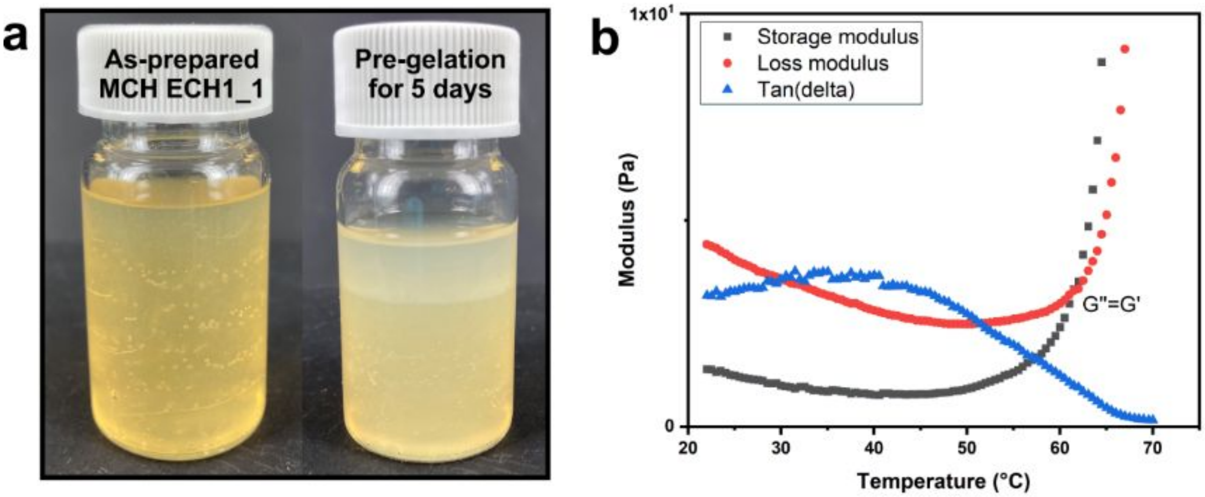

Figure S6. Digital photo of IPAMica/chitin/ECH1:1 after 5 days pre-gelation (**a**), temperature-dependent rheology of pre-gelated hydrogel of IPAMica/chitin/ECH1:1 (**b**)

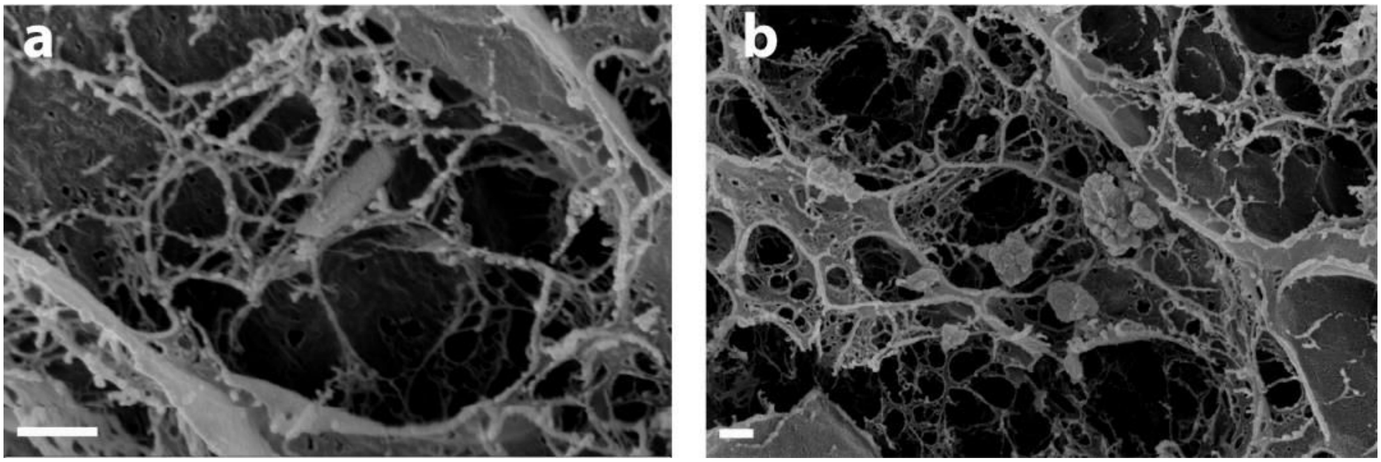

Figure S7. ECH crosslinked IPAMica/chitin hydrogel (IPAMica/chitin/ECH1:1) (**a**), entanglement of IPAMica in chitin network (**b**) (scale bar, 300 nm)

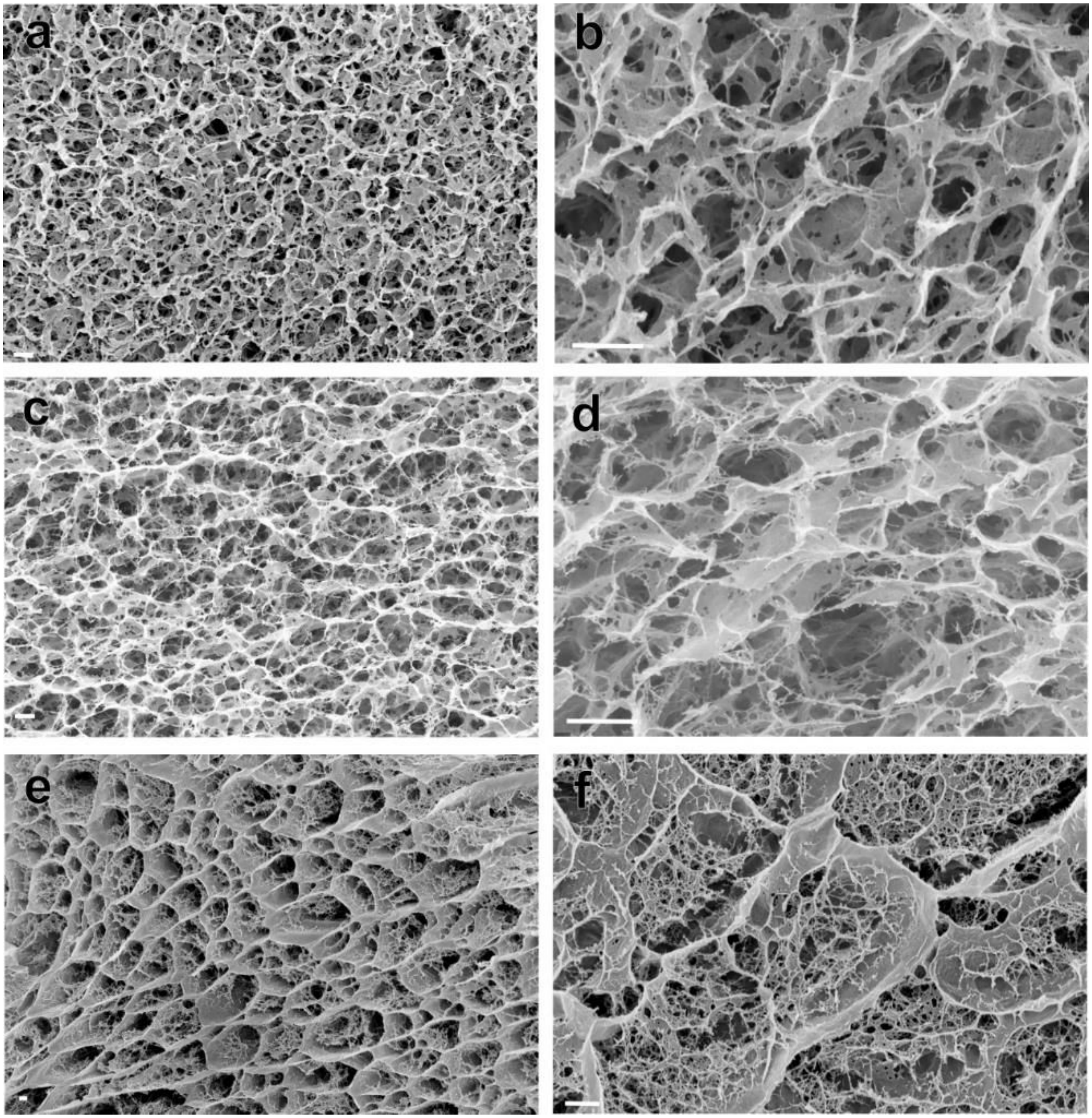

Figure S8. Cross-sectional SEM images of IPAMica/chitin (physically crosslinked) hydrogel (**a, b**), chitin/ECH (chemically crosslinked) hydrogel (**c, d**), and IPAMica/chitin/ECH1:1 (double crosslinked) hydrogel (**e, f**) (scale bar 1 μm)

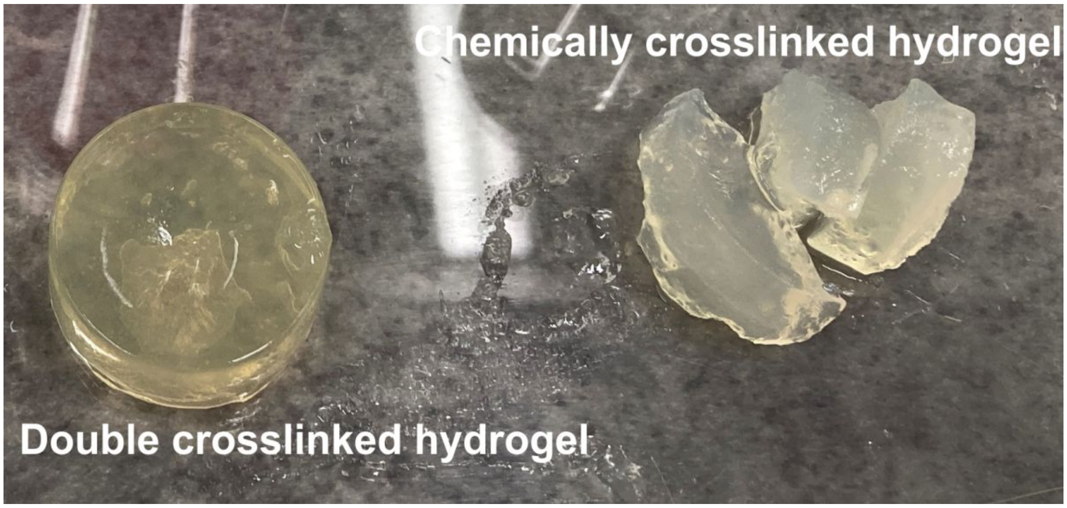

Figure S9. IPAMica/chitin/ECH1:1 (double crosslinked) hydrogel and chitin/ECH (chemically crosslinked) hydrogels cured at room temperature for 2 months

Table S3 Comparison of compressive strength of clay-chitin/chitosan-based hydrogels

Materials	Chitin or chitosan concentration (wt%)	Nanoclay content (wt%)	ECH to 1 g of solution (µL)	Strength (KPa)	Reference
HNTs/Chitin	2	80	100	59.2	[1]
MMT/Chitosan	1	1	-	0.416	[2]
**IPAMica/chitin/ECH1:1**	**3**	**-**	**8.9**	**1570**	**This work**

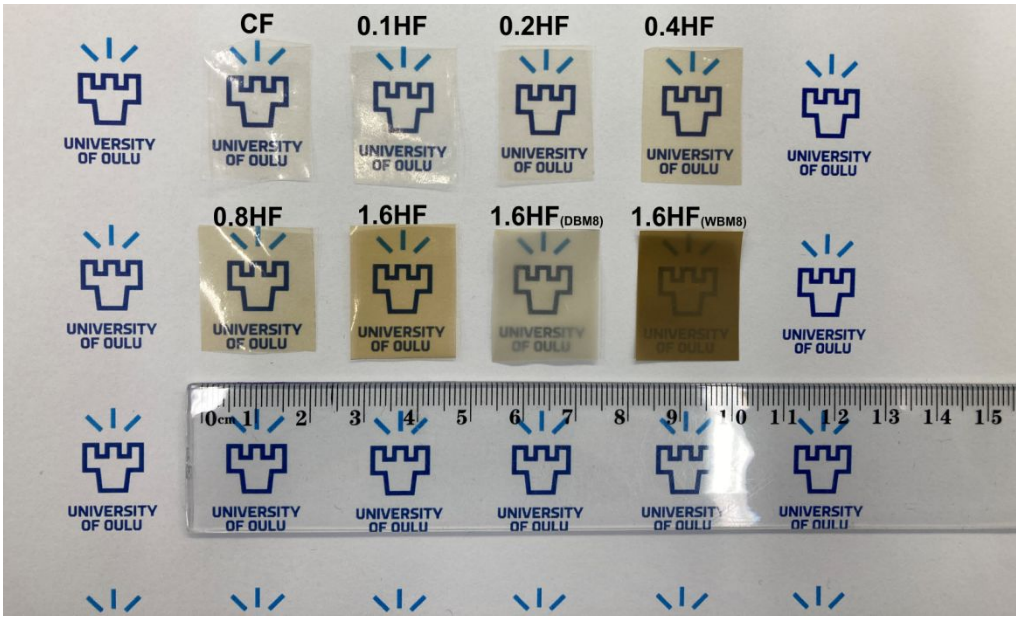

Figure S10. Digital photo of reference chitin film (CF), chitin/IPAMica hybrid films (HF) with different IPAMica content and films containing DBM8 and WBM8 (1.6HF$_{(DBM8)}$ and 1.6HF$_{(WBM8)}$)

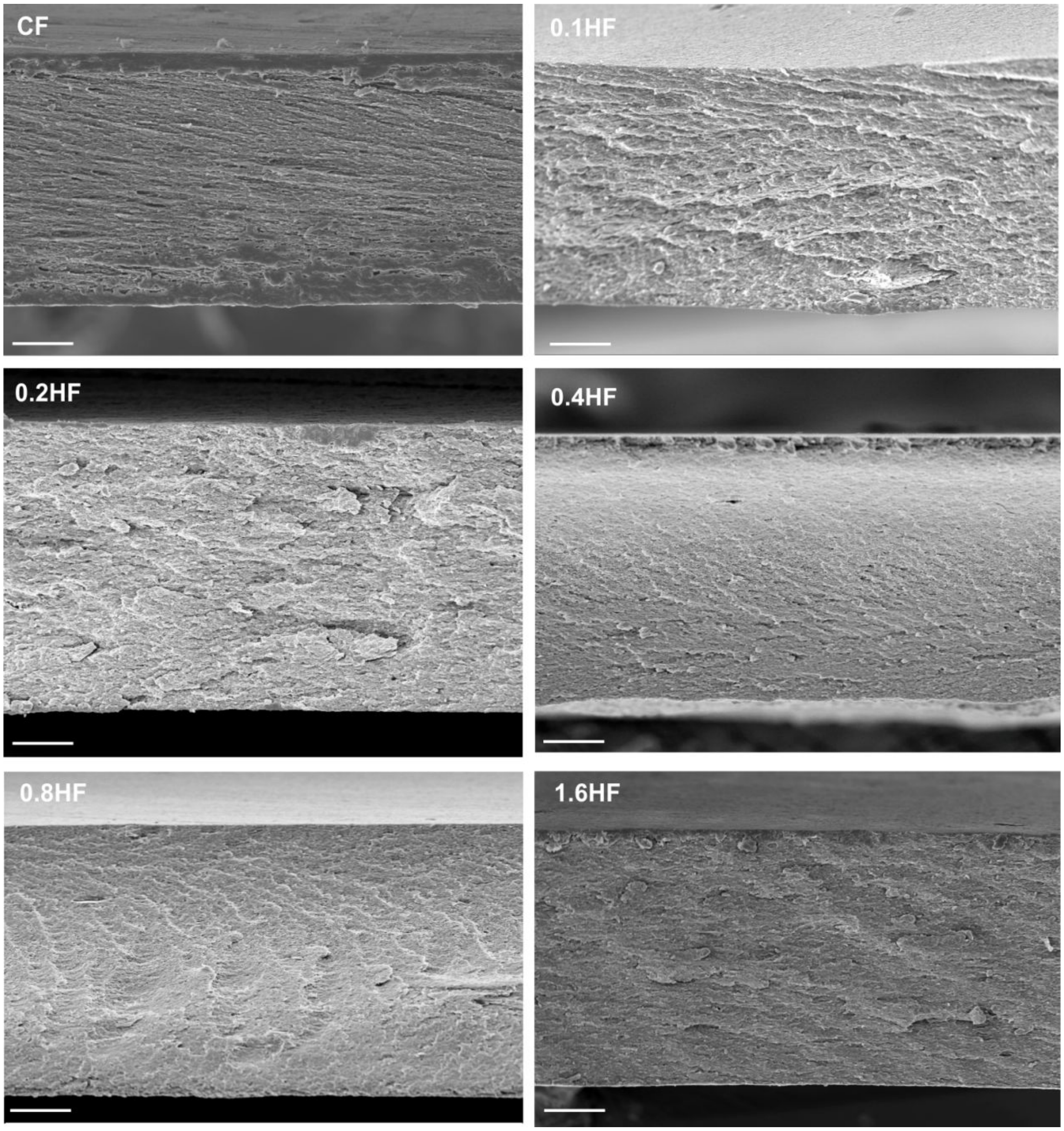

Figure S11. Cross-sectional SEM images of chitin film (CF), chitin/IPAMica hybrid film (HF) with varying IPAMica contents. Scale bars, 10 μm.

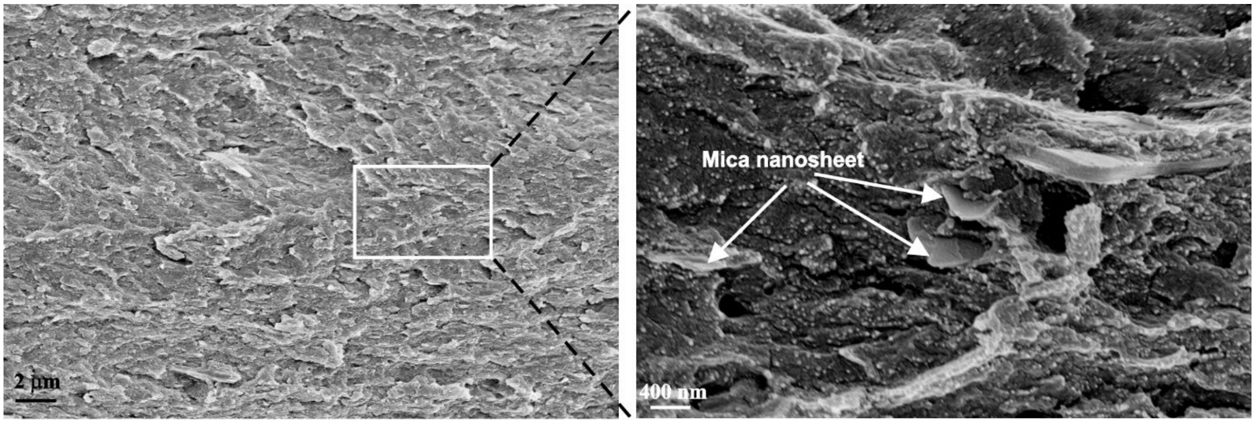

Figure S12. Cross-sectional SEM images of 1.6HF, pull-out model can be recognized

Table S4 Summary of mechanical properties of chitin/IPAMica hybrid films*

Sample abbreviation	Tensile strength	Strain	Young's modulus	Toughness
CF	39.21 MPa	56.50 %	0.85 GPa	15.64 MJ/m^3
0.1HF	43.69 MPa	24.05%	1.73 GPa	9.37 MJ/m^3
0.2HF	37.49 MPa	14.41%	1.98 GPa	3.53 MJ/m^3
0.4HF	42.32 MPa	8.74%	2.11 GPa	3.27 MJ/m^3
0.8HF	32.78 MPa	0.99%	3.54 GPa	0.41 MJ/m^3
1.6HF	29.20 MPa	37.93%	1.05 GPa	7.96 MJ/m^3

* N.B.: The values are denoted as average.

REFERENCE

(1) Liu, M.; Zhang, Y.; Li, J.; Zhou, C. Chitin-Natural Clay Nanotubes Hybrid Hydrogel. *International Journal of Biological Macromolecules* **2013**, *58*, 23–30. https://doi.org/10.1016/j.ijbiomac.2013.03.042.

(2) Tekay, E.; Aydınoğlu, D.; Şen, S. Effective Adsorption of Cr(VI) by High Strength Chitosan/Montmorillonite Composite Hydrogels Involving Spirulina Biomass/Microalgae. *Journal of Polymers and the Environment* **2019**, *27* (8), 1828–1842. https://doi.org/10.1007/s10924-019-01481-4.
